# Network Meta-Analysis of 10 Storage Mediums for Preserving Avulsed Teeth

**DOI:** 10.3389/fmed.2021.749278

**Published:** 2021-10-11

**Authors:** Na Zhang, Yuzhao Cheng, Fenglan Li, Qian Kang

**Affiliations:** Department of Oral Medicine, Shanxi Provincial People's Hospital, Taiyuan, China

**Keywords:** avulsed teeth, first aid, storage media, systematic review, network meta-analysis

## Abstract

Many storage mediums are available for the storage of avulsed teeth to preserve the viability of periodontal ligament (PDL) cells before replantation; however, it is unclear which medium is the optimal option. We performed this network meta-analysis to answer this question. A comprehensive search was conducted in PubMed, EMBASE, and the Cochrane library to capture eligible studies investigating the comparative efficacy of Hank's balanced salt solution (HBSS), aloe vera gel (AVG), oral rehydration solution (ORS), coconut water, egg white, green tea, propolis, saline, milk, and water. Statistical analysis was conducted using Review Manager v5.3 and ADDIS v1.16.8. In total, 20 RCTs involving 31 reports were included finally. Direct meta-analysis suggested that HBSS was superior to ORS, milk, saline, and water, ORS was superior to milk but inferior to coconut water and propolis, egg white was superior to milk but inferior to AVG and propolis, propolis was superior to AVG, milk, and saline, and coconut water and water was inferior to saline and milk, respectively. Network meta-analysis suggested that AVG was inferior to the other nine mediums, and propolis was superior to HBSS (SMD, −5260.24; 95% CrI, −10447.39 to −70.37) and milk (SMD, −5461.11; 95% CrI, −10574.99 to −328.51). Moreover, ranking probabilities indicated the highest probability for propolis, followed by saline, ORS, HBSS, milk, egg white, water, green tea, and AVG successively. Propolis may be the optimal media for storing avulsed teeth before replantation. However, given the availability of propolis and HBSS and the hypotonic properties of saline, ORS or milk should also be preferentially selected.

## Introduction

Avulsion or exarticulation is defined as a result of traumatic injuries, such as traffic accidents, where a tooth is dislodged from its alveolar socket (1). Published data indicate an incidence of 0.5–16% for avulsion or exarticulation (2), and it is noted that avulsion or exarticulation is regarded as one of the serious types of traumatic dental injuries (3). Ideally, an avulsed tooth is suggested to be immediately replanted in order to prevent further damage to the PDL cells from desiccation when people suffered from traumatic injuries (4, 5). However, it is not always possible to perform immediate replantation due to several factors such as life-threatening traumatic injuries, complex injury to the alveolar socket, or lack of awareness about immediate replantation (6). Therefore, an avulsed tooth is recommended to be immersed in a suitable storage media when immediate replantation can not be conducted (7). However, two key factors largely predict the prognosis of the replanted tooth, including (a) the extra-oral time of the avulsed tooth and (b) characteristics of storage media used to store the avulsed tooth prior to replantation (8–10). And it is thus critically important to select an optimal media to preserve avulsed tooth at the suitable time.

Studies suggested that ideal storage media are defined as having the ability to preserve the viability, mitogenicity, and clonogenic capacity of the PDL cells for the purpose of facilitating the healing of the replantated tooth, thereby improve the survival rate of the replantated tooth (11, 12). Up to now, numerous storage mediums, such as tap water, saliva, saline solution, milk (natural or processed type), culture media, Hank's Balanced Salt Solution (HBSS), oral rehydration solution (ORS), and coconut water, have been extensively used in practice (13, 14). In 2020, however, a systematic review and meta-analysis was conducted to compare the available mediums with milk or saliva in storing avulsed teeth prior to replantation, and the study suggested that HBSS, propolis, ORS, or cling film exhibit benefits compared to milk before replantation (7). However, which storage media may be the overall optimal selection cannot be determined in this meta-analysis because it is impossible to simultaneously estimate the comparative efficacy of more than two comparisons at a time with the conventional head-to-head meta-analysis.

Fortunately, network meta-analysis, as an expanded method of pair-wise meta-analysis, has been developed and widely used to simultaneously pool multiple evidence, including direct and indirect evidence to generate more comprehensive, reliable, and robust findings, which addresses those shortages faced by traditional head-to-head meta-analysis (15). Considering the advantages, we therefore performed the current network meta-analysis to estimate the comparative efficacy of 10s common storage mediums—HBSS, aloe vera gel (AVG), ORS, coconut water, egg white, green tea, propolis, saline, milk, and water—in preserving the viability of PDL cells before replantation in order to further determine which medium should be preferentially selected to store avulsed teeth in clinical practice.

## Methods

We developed the methodological framework of the present network meta-analysis in line with the recommendations suggested by the Cochrane Collaboration (16). After completing statistical analysis of the network meta-analysis, we reported results according to the Preferred Reporting Items for Systematic Reviews and Meta-analyses (PRISMA) for Network Meta-Analysis (PRISMA-NMA) Checklist (17, 18). The filled PRISMA checklist can be seen in Supplementary Table 1. Our network meta-analysis does not require ethical approval and patient's informed consent because we performed all statistical analyses using published data.

### Identification of Studies

In our network meta-analysis, two independent reviewers were assigned to electronically search the eligible databases in order to match eligible studies. In total, the three databases were PubMed, EMBASE, and the Cochrane Library. The time limit of our search was between their inception and April 2021. A systematic search method of combining medical subject heading (MeSH) with text words was used to develop the basic search strategy, and then a unique strategy was developed by modifying the basic search strategy according to the criteria of an individual database. The details of all search strategies can be found in Supplementary Table 2. Only studies published in the English language were considered to meet our inclusion criteria; however, we did not impose the restriction of publication status. Additionally, we also utilized the hand-check method to review the references of all included studies and published meta-analyses to add additional eligible studies. Any disagreements on the identification of studies were resolved through consulting a third senior reviewer.

### Selection Criteria

According to the previous meta-analysis (7), we designed the following selection criteria: (a) adults and children with an avulsed or extracted permanent tooth; (b) any comparison that was constructed based on at least 2 of the 10 common storage mediums—HBSS, AVG, ORS, coconut water, egg white, green tea, propolis, saline, milk, and water—was reported; and (c) only randomized controlled trial (RCT) was considered to be eligible. Moreover, we only considered the latest study with more adequate information when a series of studies were published by the same research team based on the same population. Studies were excluded if they covered at least one of the following criteria: (a) they used cultured cells of the PDL or extracted animal teeth; (b) storage media was unavailable to laypeople; (c) they only investigated financial costs of storage media; and (d) they were reviews, editorials, letters, case reports, conference abstracts, and cell and animal studies. Two reviewers independently completed the process of selecting studies. Any disagreements about the selection of studies were resolved through consulting a third senior reviewer.

### Data Extraction

We assigned two independent reviewers to extract essential information using a data extraction sheet: basic information of studies such as the name of the first author, publication year, and country of the corresponding author, the basic information of research target, basic information of medium, outcomes, and details of the risk of bias. Any disagreements about data extraction were resolved through consulting a third senior reviewer.

### Outcomes of Interesting

In the current network meta-analysis, we simultaneously considered the following outcomes, including the cell viability, pain, malfunction, color of the tooth, success of the replantation, and infection rate. We defined cell viability as the primary outcome and defined the remaining outcomes as the secondary outcome.

### Quality Assessment

As eligible studies in the present network meta-analysis are designed to focus on avulsed teeth and investigate objective outcomes, we deleted two items in the assessment of the blind method to modify the Cochrane Risk of Bias assessment tool (19). Two independent reviewers were then assigned to assess the quality of an individual study from the following five domains: random sequence generation; allocation concealment; incomplete outcome data; selective reporting; and other bias. We labeled a study as having a low risk of bias if all domains were fulfilled. We labeled a study as having a high risk of bias if more than one of all domains were not fulfilled. A study was labeled with unclear risk of bias when there was not sufficient information for determination. Any disagreements about the risk of bias assessment were resolved through consulting a third senior reviewer.

### Statistical Analysis

In the current study, we simultaneously performed a head-to-head meta-analysis and network meta-analysis in order to investigate the comparative efficacy of 10 common storage mediums in storing avulsed teeth prior to replantation.

For the conventional pair-wise meta-analysis, we used Cochrane Review Manager (RevMan) software version 5.3 (The Nordic Cochrane Centre, the Cochrane Collaboration, Copenhagen, 2014) to calculate all estimates based on the random-effects model. Because the eligible studies only reported cell viability as a continuous variable, we calculated standard mean difference (SMD) with a 95% confidence interval (CI) to express all pooled results. We firstly qualitatively inspected the heterogeneity across studies using Cochrane Q statistic (*p*-value), and then we used *I*^2^ statistic to quantitatively estimate the proportion of heterogeneity except for random error. If *I*^2^ <50% and *P* > 0.1, studies were considered to be homogeneous. In contrast, studies were defined as heterogeneous when *I*^2^ ≥ 50% and *P* < 0.1.

After completing conventional pair-wise meta-analysis, we then performed Bayesian network analysis with the Aggregate Data Drug Information System software (ADDIS V.1.16.8, Drugis, Groningen, NL), which was designed to calculate all estimates based on the Markov Chain Monte Carlo (MCMC) simulation method (20). We calculated SMD with a 95% credible interval (CrI) to express results estimated from network meta-analysis. All results were estimated from the random-effects and consistency models if the node split method (21) did not indicate an inconsistency between direct and indirect effects based on the following parameters: (a) 4 chains; (b) 20,000 tuning iterations; (c) 50,000 simulation iterations; (d) thinning interval of 10; (e) 10,000 inference samples; and (f) variance scaling factor of 2.5. In contrast, we used random-effects and inconsistency models to estimate results if the node split method (21) indicated inconsistencies between direct and indirect effects. Moreover, we used the Brooks-Gelman-Rubin method to evaluate the convergence of iteration based on the potential scale reduction factor (PSRF). A PSRF of closing to 1 indicates a good convergence, while a PSRF of <1.2 was considered acceptable. Finally, we used Microsoft Excel to generate ranking probabilities of 10 common storage mediums based on the results calumniated from ADDIS software.

### Publication Bias

We generated funnel plots regarding the comparison of HBSS and milk to qualitatively inspect the possibility of the presence of publication bias because the accumulated eligible numbers of reports for this comparison were more than 10 (22).

## Results

### Identification and Selection of Studies

**Figure 1:**
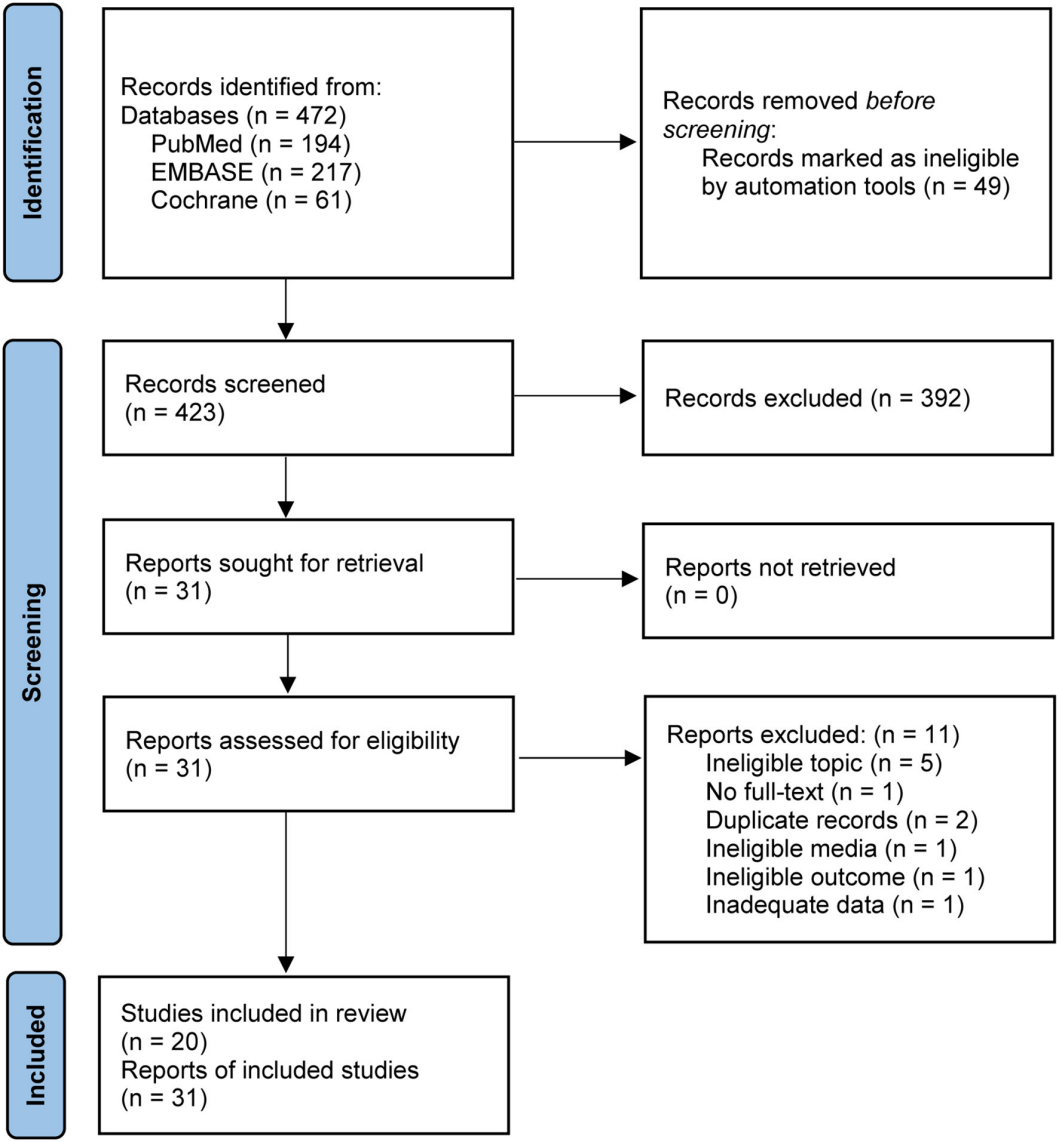
Flow diagram of searching and selecting studies. We searched PubMed, EMBASE, and the Cochrane Library to determine potentially eligible studies.

We identified 472 potentially eligible records by searching PubMed, EMBASE, and the Cochrane Library from their inception to April 2021. In total, 423 unique records were retained after removing 49 duplicate records. After initially checking the eligibility of remaining records based on title and abstract, 392 ineligible records were deleted. We obtained 31 full texts to further verify their eligibility. After evaluating the full texts, 11 studies were excluded due to six reasons: ineligible topic (*n* = 5), no full text available (*n* = 1), duplicate records (*n* = 2), ineligible media (*n* = 1), ineligible outcome (*n* = 1), and inadequate data (*n* = 1), and then 20 eligible studies (6, 23–41) including 31 reports were considered to be eligible for our inclusion criteria. The identification and selection of studies were Figure 1.

### Basic Characteristics of Eligible Studies

These 20 eligible studies were published between 1998 and 2019. The total number of avulsed teeth in the individual studies was between 30 and 120, with the overall total being 1,048. The majority of studies (RCTs), 11 (55.0%), were carried out in India, 5 (25.0%) were carried out in Iran, 2 (10.0%) in the USA, 1 (5.0%) in China, and 1 (5.0%) was carried out in Thailand. All eligible studies only reported the number or percentage of viable PDL cells to evaluate cell viability, however secondary outcome was not reported by all eligible studies. Details of these 20 eligible studies were shown in Table 1.

**Table 1:** Basic characteristics of included 20 studies.

**References**	**Country**	**Research target**	**Details of comparisons**	**Details of immersion**	**Outcomes**
Abraham et al. (6)	India	40 freshly extracted human premolars	HBSS (*n =* 10), AVG (*n =* 10) or milk (*n =* 10)	Drying for 30 min followed by 45 min immersion	Number of viable PDL cells
Ahangari et al. (23)	Iran	60 extracted teeth	propolis 10% (*n =* 10), propolis 50% (*n =* 10), HBSS (*n =* 10), egg white (*n =* 10), milk (*n =* 10)	Drying for 30 min followed by 1–3 h immersion	Percentage of viable PDL cells
Babaji et al. (24)	India	50 extracted teeth	HBSS (*n =* 10), propolis 50% (*n =* 10), AVG (*n =* 10)	n.r.	Number of viable PDL cells
Chen et al. (25)	China	30 freshly extracted single-rooted teeth	green tea (*n =* 10), HBSS (*n =* 10), or milk (*n =* 10)	2 h immersion	Percentage of viable PDL cells
D'Costa et al. (26)	India	40 extracted sound human premolars	coconut water (*n =* 10), saline (*n =* 10), or milk (*n =* 10)	Drying for 30 min followed by 45 min immersion	Percentage of viable PDL cells
Doyle et al. (27)	USA	49 freshly extracted human teeth	HBSS (*n =* 15) or milk (*n =* 15)	Drying for 30, 60, or 90 min followed by 15 min immersion	Percentage of viable PDL cells
Ghasempour et al. (28)	Iran	54 extracted human teeth	water (n = 18), HBSS (n = 18), or green tea (n = 18)	1, 3, 15 h immersion	Number of viable PDL cells
Gopikrishna et al. (29)	India	55 freshly extracted human teeth	coconut water (*n =* 15), HBSS (*n =* 15), or milk (*n =* 15)	Drying for 30 min followed by 45 min immersion	Number of viable PDL cells
Khademi et al. (30)	Iran	100 extracted permanent premolars	egg white (*n =* 25), HBSS (*n =* 25), water (*n =* 25), or milk (*n =* 25)	1, 2, 4, 8, or 12 h immersion	Percentage of viable PDL cells
Kokkali et al. (31)	India	55 freshly extracted premolars	coconut water (*n =* 15), butter milk (*n =* 15), or milk (*n =* 15)	Drying for 30 min followed by 45 min immersion	Number of viable PDL cells
Martin et al. (32)	USA	70 freshly extracted single-rooted teeth	saline (*n =* 12), HBSS (*n =* 12), propolis 50% (*n =* 12), propolis 100% (*n =* 12), or milk (*n =* 12)	Drying for 30 min followed by 45 min immersion	Number of viable PDL cells
Nabavizadeh et al. (33)	Iran	40 freshly extracted single-rooted teeth	HBSS (*n =* 10), or milk (2.5% fat) (*n =* 10)	Drying for 30 min followed by 45 min immersion	Percentage of viable PDL cells
Prueksakorn et al. (34)	Thailand	96 closed-root-apex premolars	propolis (*n =* 10), HBSS (*n =* 10), or milk (*n =* 10)	Drying for 30 min followed by 180 min immersion	Percentage of viable PDL cells
Rajendran et al. (35)	India	30 freshly extracted human teeth	ORS (*n =* 10), HBSS (*n =* 10), or milk (*n =* 10)	Drying for 30 min followed by 45 min immersion	Number of viable PDL cells
Saini et al. (36)	India	69 freshly extracted non-carious premolars	coconut water (*n* = 23), probiotic milk (*n* = 23), or HBSS (*n* = 23)	Drying for 20 min followed by 30 min immersion	Number of viable PDL cells
Sanghavi et al. (37)	India	40 freshly extracted teeth	coconut water (*n =* 10), propolis 50% (*n =* 10), or ORS (*n =* 10)	Drying for 30 min followed by 30 min immersion	Number of viable PDL cells
Sharma et al. (39)	India	45 non-carious human premolar teeth	AVG (*n =* 15), egg white (*n =* 15), or milk (3.0% fat) (*n =* 15)	Drying for 15 min followed by 30 min immersion	Percentage of viable PDL cells
Sharma et al. (38)	India	45 non-carious human mature premolars	egg white (*n =* 15), or milk (3.0% fat) (*n =* 15)	Drying for 15 min followed by 30 min immersion	Percentage of viable PDL cells
Subramaniam et al. (40)	India	120 sound- and caries-free premolars	HBSS (*n =* 10), ORS (*n =* 10), milk (3.0% fat) (*n =* 10)	Drying for 30 or 60 min followed by 45 or 90 min immersion	Number of viable PDL cells
Talebi et al. (41)	Iran	60 mature, healthy extracted premolars	HBSS (*n =* 15), water (*n =* 15), or milk (*n =* 15)	Drying for 30 min followed by 1, 3, 6, or 24 h immersion	Percentage of viable PDL cells

*HBSS, Hank's Balanced Salt Solution; PDL, periodontal ligament; AVG, aloe vera gel; n.r., not reported; ORS, oral rehydration solution*.

### Risk of Bias

Among the 20 included studies, only three studies (31, 38, 39) definitively reported the methods of generating random sequence, none of the studies (6, 23–41) reported the details of performing allocation concealment, and all studies were rated as low risk of bias in incomplete data, selective reporting, and other bias domains. Overall, the level of risk of bias among all studies was considered to be moderate. The summary of the risk of bias was delineated in Supplementary Table 3.

### Evidence Structure

**Figure 2:**
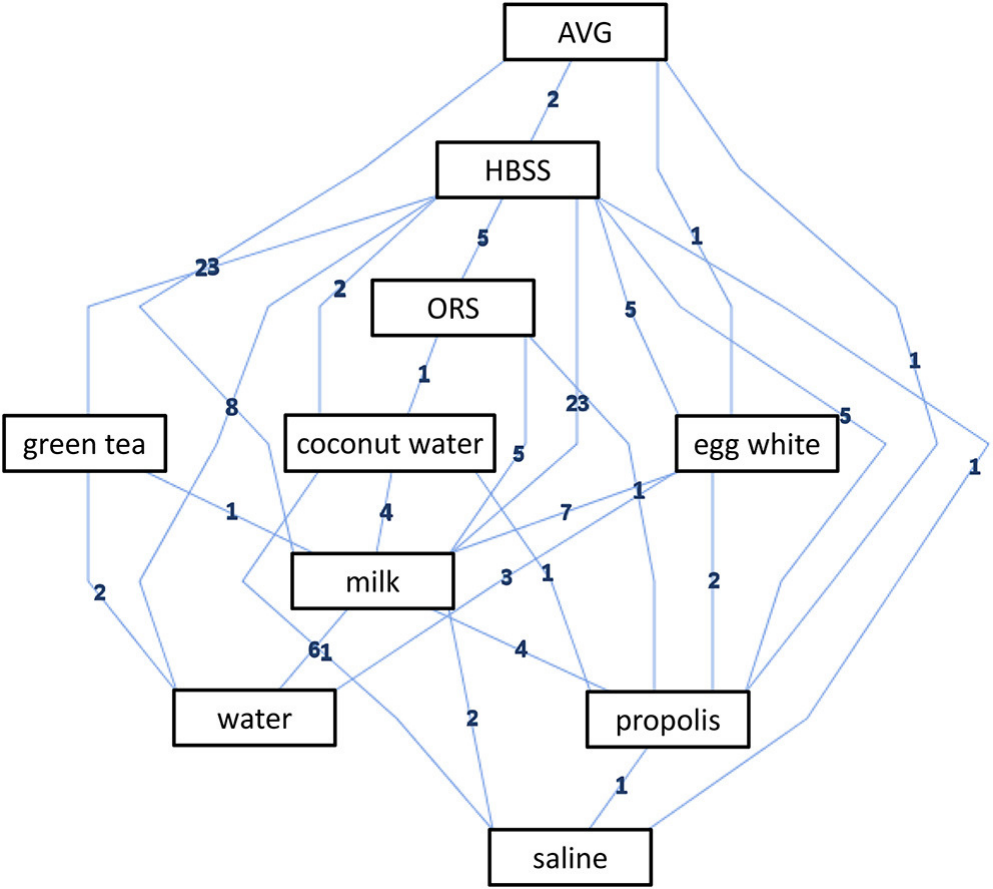
Evidence structure of cell viability.

In the current network meta-analysis, all eligible studies only reported the data of cell viability. We constructed the evidence plot of cell viability based on ADISS software. Evidence structure indicated that the comparison between HBSS and milk was supported by 23 pieces of direct evidence, the comparison between HBSS and water was supported by eight pieces of direct evidence, and the comparison between egg white and milk was supported by pieces of seven direct evidence. Details of the evidence structure were displayed in Figure 2.

### Direct Meta-Analysis

Among 31 reports from 20 eligible studies, 5, 23, 1, and 5 report(s) compared HBSS with ORS, milk, saline, and water, respectively, and pooled results suggested that HBSS was superior to ORS (SMD, 1.03; 95% CI, 0.26–1.80), milk (SMD, 2.63; 95% CI, 1.62–3.64), saline (SMD, 1.62; 95% CI, 0.72–2.52), and water (SMD, 4.23; 95% CI, 3.04–5.41) in preserving the viability of PDL cells (Supplementary Table 4).

Among 31 reports from 20 eligible studies, 1, 1, and 5 report(s) compared ORS with coconut water, propolis, and milk, respectively, and pooled results suggested that ORS was superior to milk (SMD, 3.40; 95% CI, 1.10–5.70), but was inferior to coconut water (SMD, −3.78; 95% CI, −5.22 to −2.34) and propolis (SMD, −3.66; 95% CI, −5.07 to −2.25) in preserving the viability of PDL cells (Supplementary Table 4).

Among 31 reports from 20 eligible studies, 1 report compared AVG with egg white, and the pooled result suggested that AVG was superior to egg white (SMD, 1.68; 95% CI, 0.86–2.49) in preserving the viability of PDL cells (Supplementary Table 4). Moreover, six reports also compared egg white with milk, and meta-analysis suggested a beneficial result for egg white (SMD, 5.84; 95% CI, 2.81–8.88) in preserving the viability of PDL cells (Supplementary Table 4).

Among 31 reports from 20 eligible studies, 1, 2, 4. and 1 report(s) compared propolis with AVG (SMD, 14931.26; 95% CI, 10304.10–19558.42), egg white (SMD, 2.25; 95% CI, 1.00–3.49), milk (SMD, 1.88; 95% CI, 1.28–2.48), and water (SMD, 1.33; 95% CI, 0.58–2.07), and pooled results suggested that propolis was superior to all comparators pointed out above in preserving the viability of PDL cells (Supplementary Table 4).

Among 31 reports from 20 eligible studies, 1 and 3 report(s) compared milk with saline (SMD, −20.38; 95% CI, −26.75 to −14.01) and water (SMD, −2.49; 95% CI, −3.24 to −1.75), respectively, and pooled suggested that milk was superior to that two compared storage medium in preserving the viability of PDL cells (Supplementary Table 4).

### Network Meta-Analysis of Cell Viability

We also performed a network meta-analysis to further investigate the comparative efficacy of these 10 common storage mediums. However, we first adopted the split-node method to check the network inconsistency in the current network meta-analysis, and the result indicated an inconsistency between direct and indirect effects when HBSS compared to AVG, AVG compared to milk, AVG compared to propolis, and propolis compared to milk (Table 2). We, therefore, calculated the results of these comparisons based on the inconsistency model; however, the results of the remaining comparisons were estimated based on the consistency model.

**Table 2:** Inconsistency examination based on node split method.

**Name**	**Direct effect**	**Indirect effect**	**Overall effect**	***P*-value**
HBSS, ORS	−77.93 (−6,612.47, 6,563.79)	2,361.01 (−11,247.60, 15,516.61)	336.87 (−4,897.76, 5,618.32)	0.74
HBSS, AVG	−18,290.02 (−25,437.99, −11,449.03)	651.00 (−8,551.49, 9,951.22)	−15,278.64 (−22,855.64, −7,974.56)	0.00
HBSS, coconut water	8.93 (−10,180.83, 10,018.89)	−1,598.58 (−9,564.76, 6,632.89)	−839.17 (−7,050.78, 5,208.95)	0.82
HBSS, egg white	−19.14 (−6,332.96, 6,332.29)	−4,875.30 (−14,930.99, 4,998.83)	−552.91 (−5,612.35, 4,428.42)	0.41
HBSS, milk	−26.97 (−3,003.50, 3,006.96)	−958.53 (−8,707.98, 7,146.11)	−77.90 (−3,013.36, 2,850.95)	0.82
HBSS, propolis	4,602.68 (−396.21, 9675.86)	−1,276.98 (−11,799.19, 9,197.28)	5,113.88 (−113.62, 10,312.38)	0.31
HBSS, saline	−110.59 (−14,366.10, 14,088.35)	−716.76 (−13,717.21, 12,138.74)	639.91 (−8,333.11, 9,487.52)	0.95
ORS, coconut water	−82.53 (−14,308.39, 14,407.71)	−2,625.74 (−11,005.83, 5,949.56)	−1,160.28 (−8,679.51, 6,237.70)	0.75
ORS, milk	−8.05 (−6,564.63, 6,431.65)	−607.49 (−7,265.61, 5,944.64)	−451.63 (−5,703.02, 4,833.60)	0.90
ORS, propolis	76.09 (−14,116.26, 14,251.80)	6,370.02 (−1,227.06, 13,785.89)	4,743.49 (−2,125.99, 1,1604.15)	0.43
AVG, egg white	−82.87 (−14,278.49, 14,068.75)	17,293.05 (8,329.59, 26,327.99)	14,753.37 (6,824.75, 22,899.00)	0.04
AVG, milk	334.14 (−9,301.35, 9,979.32)	19,181.28 (11,069.48, 27,168.38)	15,163.14 (7,907.96, 22,625.43)	0.01
AVG, propolis	59,002.41 (49,655.51, 68,354.16)	9,874.75 (3,853.38, 15,943.98)	20,444.81 (12,282.81, 28,653.47)	0.00
Coconut water, milk	2,954.68 (−5,245.05, 11,127.55)	−1,442.77 (−9,483.62, 6,307.75)	719.45 (−5,247.56, 6,869.92)	0.42
Coconut water, propolis	−172.58 (−14,065.92, 14,335.99)	7,762.31 (−230.35, 15,896.11)	5,982.58 (−1,347.46, 13,236.58)	0.34
Coconut water, saline	−26.05 (−14,159.62, 14,581.49)	2,595.47 (−10,935.30, 15,708.10)	1,498.98 (−8,075.40, 11,079.12)	0.79
Egg white, milk	−74.50 (−5,713.86, 5,759.55)	1,697.56 (−4,841.14, 8,366.94)	499.27 (−4,435.94, 5,323.61)	0.69
Egg white, propolis	39.40 (−9,600.07, 9,580.72)	9,490.21 (1,766.80, 17,509.08)	5,708.58 (−697.14, 12,080.77)	0.13
Green tea, milk	84.11 (−14,544.42, 14,313.78)	−113.84 (−8,440.05, 8,115.15)	−70.20 (−7,721.08, 7,538.14)	0.99
Green tea, water	−277.15 (−10,233.41, 10,107.61)	−101.70 (−10,818.78, 10,688.62)	−149.22 (−8,052.66, 7,969.11)	1.00
Milk, propolis	44.65 (−6,198.80, 6,317.83)	17,987.50 (9,612.13, 26,400.94)	5,171.85 (59.51, 10,556.95)	0.00
Milk, water	−43.24 (−8,090.10, 8,396.44)	−78.91 (−10,154.25, 10,030.47)	−33.54 (−5,996.11, 6,042.95)	1.00
Propolis, saline	−163.10 (−14,344.89, 14,598.58)	−5,778.18 (−19,570.59, 8,053.41)	−4,470.99 (−14,059.98, 4,956.82)	0.57

*HBSS, Hank's balanced salt solution; ORS, oral rehydration solution; AVG, aloe vera gel*.

Pooled result from network meta-analysis based on the consistency or inconsistency model suggested that AVG was inferior to all other storage medium in preserving the viability of PDL cells (Table 3). Moreover, propolis was superior to HBSS (SMD, −5,260.24; 95% CrI, −10,447.39 to −70.37) and milk (SMD, −5,461.11; 95% CrI, −10,574.99 to −328.51) in preserving the viability of PDL cells (Table 3). All consequences of network meta-analysis of cell viability were summarized in Table 3.

**Table 3:** Network meta-analysis of 10 common storage mediums in terms of cell viability.

**AVG**									
**−15299.73** **(−22386.52,** **−7989.93)**	HBSS								
**−15646.04** **(−24362.96,** **−7124.52)**	−375.95 (−5528.99, 4730.81)	ORS							
**−15385.37** **(−24537.64,** **−6276.55)**	−91.72 (−6158.79, 5748.93)	295.97 (−6813.97, 7609.39)	coconut water						
**−14705.67** **(−22708.50,** **−6559.65)**	629.31 (−4448.29, 5599.63)	998.31 (−5806.91, 7739.54)	738.26 (−6848.47, 8194.08)	egg white					
**−15292.02** **(−25400.82,** **−5151.07)**	34.75 (−7245.54, 7411.31)	422.82 (−8315.01, 9160.11)	80.90 (−9068.72, 9471.34)	−590.31 (−9346.76, 8113.65)	green tea				
**−15135.30** **(−22307.65,** **−7744.29)**	222.77 (−2640.44, 3024.31)	597.54 (−4481.99, 5679.43)	281.09 (−5373.01, 6224.11)	−399.78 (−5272.07, 4551.45)	187.41 (−7299.94, 7641.23)	milk			
**−20560.22** **(−28653.01,** **−12470.09)**	**−5260.24** **(−10447.39,** **−70.37)**	−4883.93 (−11464.31, 1732.43)	−5188.44 (−12211.30, 2094.45)	−5897.86 (−12181.97, 527.49)	−5292.50 (−14208.58, 3524.63)	**−5461.11** **(−10574.99,** **−328.51)**	propolis		
**−16088.77** **(−26938.56,** **−5138.69)**	−775.95 (−9363.82, 7690.17)	−398.56 (−10138.83, 9289.87)	−711.10 (−9985.56, 8545.66)	−1375.27 (−11079.71, 8284.10)	−855.26 (−12085.56, 10498.84)	−1010.29 (−9537.65, 7331.44)	4444.53 (−4708.44, 13522.42)	saline	
**−15086.31** **(−24070.09,** **−5944.27)**	163.62 (−5410.97, 6004.25)	557.97 (−6894.47, 8095.69)	320.36 (−7816.85, 8201.50)	−473.50 (−7797.49, 7155.23)	179.46 (−7746.46, 8086.86)	−42.84 (−5870.52, 6057.72)	5389.76 (−1908.47, 13000.35)	943.14 (−8998.53, 11347.91)	water

*HBSS, Hank's balanced salt solution; ORS, oral rehydration solution; AVG, aloe vera gel. The bold values mean statistical significance*.

### Ranking of 10 Common Storage Mediums in Terms of Cell Viability

**Figure 3:**
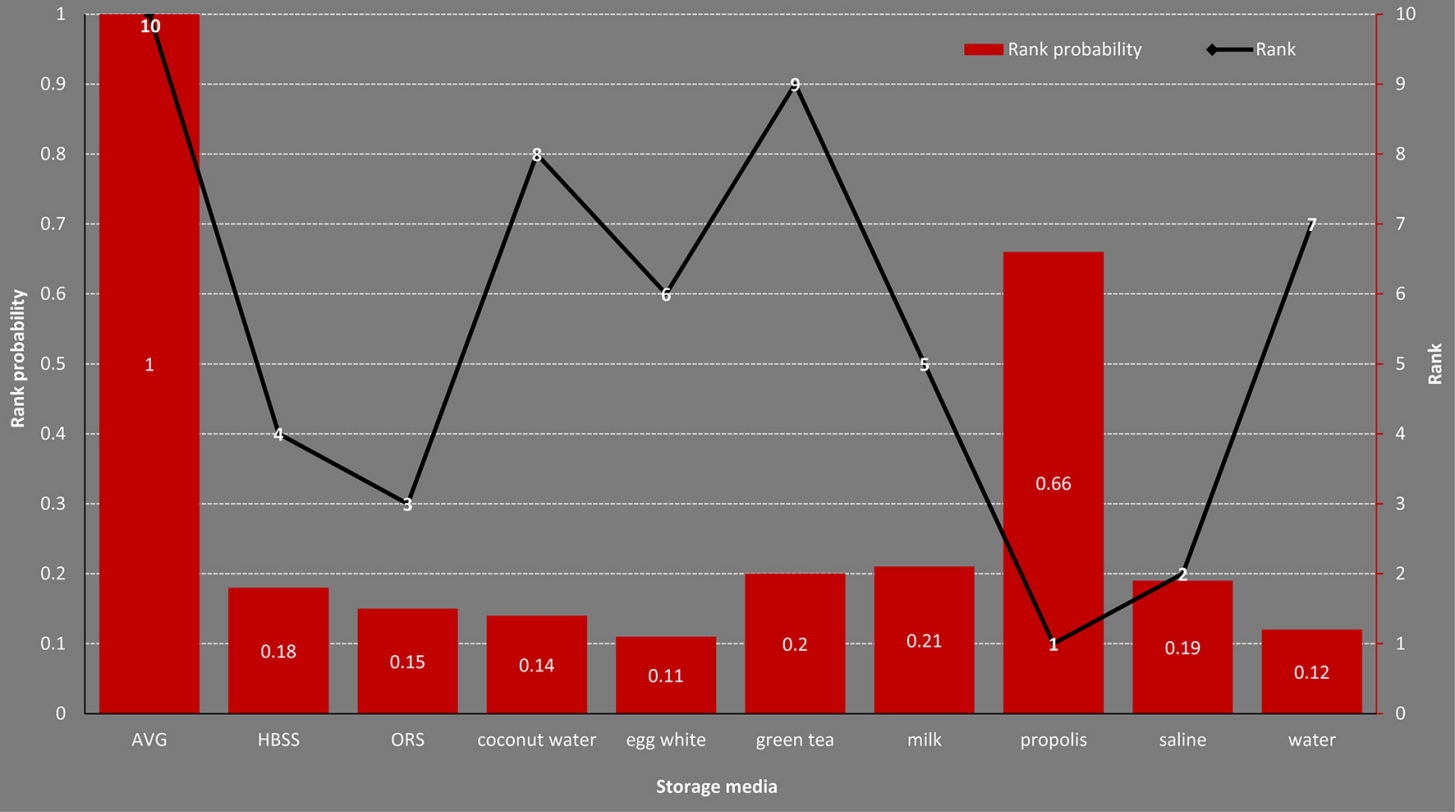
Rank probability of all storage mediums for cell viability.

We generated ranking probabilities of all storage mediums in terms of cell viability (Supplementary Table 5). Results indicated that propolis had the highest probability of being placed first as storage media, followed by saline, ORS, HBSS, milk, egg white, water, coconut water, green tea, and AVG. The plot of rankings of all storage mediums was delineated in Figure 3.

### Publication Bias

In this network meta-analysis, the accumulated number of comparisons between HBSS and milk was more than 10, with the total number being 23 reports, and thus we drew the funnel plot to qualitatively inspect whether the presence of publication bias or not. The funnel plot did do not provide evidence of publication bias (Supplementary Figure 1).

## Discussion

Avulsion or exarticulation has been regarded to be one of the most serious types of traumatic dental injuries (42). Although various storage mediums such as milk, HBSS, and coconut water have been used to store avulsed teeth for the purpose of preserving the viability of PDL cells prior to replantation (13), and a systematic review and meta-analysis has also been conducted to investigate the comparative efficacy between the various storage medium and milk (7), no definitive conclusion about which storage media should be the optimal option for persevering viability of PDL cells prior to replantation has been generated currently. Our network meta-analysis firstly investigates the comparative efficacy of 10 common storage mediums and suggested that propolis is better than AVG, HBSS, and milk in preserving the viability of PDL cells as a storage media. Meanwhile, our network meta-analysis also find that AVG should not be recommended to store avulsed teeth because it is inferior to the other nine storage media in preserving the viability of PDL cells prior to replantation. Moreover, results based on ranking probabilities also indicated that propolis should be preferentially recommended as a storage media because it is at the first rank out of the ten common mediums investigated.

To date, only one study to investigate the comparative efficacy between the available storage mediums and cow's milk using conventional pair-wise meta-analysis (7). In the previous meta-analysis, the authors included 33 primary studies involving three study designs including RCT, prospective cohort study, and non-RCT for the final analysis, and quantitative results indicated that HBSS, ORS, or propolis solution was superior to cow's milk in preserving cell viability. Although the previous meta-analysis provides several valuable information for storing avulsed teeth prior to replantation, which media may be the optimal option is not still answered. Compared to the previous meta-analysis, our network meta-analysis has three following strengths: (a) we only considered RCT to be eligible for inclusion criteria and thus generated more reliable results, (b) we simultaneously investigated the comparative efficacy between any two storage medium, which were from 10 common mediums, and they thus provided a database for ranking all mediums, and (c) we ranked 10 common storage medium, which aids in making definitive recommendations for decision making in clinical practice.

Propolis is a product from yellowish to brownish resinous, which has anti-bacterial and anti-inflammatory properties which benefit to preserve the viability of PDL cells because propolis contains many biologically active compounds. As a result, propolis has been used to store avulsed teeth as the storage media in practice, and it was ranked in first place among 10 common mediums in the current network meta-analysis. However, two major aspects must be considered when we select a suitable storage media to temporarily store an avulsed tooth prior to replantation: (a) real settings of experiencing a traumatic event and (b) availability of products for making storage media (7). Unfortunately, propolis can not be easily accessed in most low- and middle-income countries or only can be hard accessed from commercial products (7). Moreover, saline solution was not recommended or the storage of an avulsed tooth because it is deficient in essential nutrients such as magnesium and glucose that are essential to the normal metabolism of PDL cells, and, more importantly, is that the hypotonic properties of saline solution will accelerate cellular lysis (30). Meanwhile, HBSS was also not commonly available at common places of commonly occurring traumatic injuries such as schools and homes (10). It is exciting that, however, ORS can be prepared based on local ingredients and has been recommended to store an avulsed tooth in rural and remote regions (43). Therefore, if propolis or HBSS is not available for use, other suboptimal storage mediums such as ORS and milk may be preferentially considered.

Regardless of the fact that the current network meta-analysis had several strengths as introduced above, some limitations also must be further interpreted. First, we generally defined butter or probiotic milk as milk rather than unique storage media due to limited data. Second, we did not classify propolis into unique sub-categories depending on the different concentrations. Third, methods of immersion in all eligible studies were variable, which may impair the robustness of our pooled results. Fourth, the eligible studies only reported the viability of PDL cells, other outcomes such as pain, malfunction, the color of the tooth, the success of the replantation, and infection rate were not reported. Fifth, most of the eligible studies were conducted in India and Iran, and our findings should be cautiously interpreted when translated into other settings.

## Conclusions

Based on limited available evidence, we conclude that propolis may be the preferred storage media for storing avulsed teeth for the purpose of preserving the viability of PDL cells before replantation when it is available to actual settings. However, given the availability of propolis and HBSS in real settings of occurring traumatic injuries and the hypotonic properties of saline solution, ORS or milk should also be preferentially selected to store an avulsed tooth as a media. Moreover, we also suggest performing more high-quality studies in order to accurately determine the optimal storage media based on the more robust and reliable evidence base.

## Author Contributions

NZ contributed to the conception, design, data acquisition and interpretation, and drafted and critically revised the manuscript. YC contributed to the conception, design, data acquisition and interpretation, performed all statistical analyses, and drafted and critically revised the manuscript. FL and QK contributed to the conception and design and critically revised the manuscript. All authors gave their final approval and agree to be accountable for all aspects of the work.

## Conflict of Interest

The authors declare that the research was conducted in the absence of any commercial or financial relationships that could be construed as a potential conflict of interest.

## Publisher's Note

All claims expressed in this article are solely those of the authors and do not necessarily represent those of their affiliated organizations, or those of the publisher, the editors and the reviewers. Any product that may be evaluated in this article, or claim that may be made by its manufacturer, is not guaranteed or endorsed by the publisher.
